# The E6AP Binding Pocket of the HPV16 E6 Oncoprotein Provides a Docking Site for a Small Inhibitory Peptide Unrelated to E6AP, Indicating Druggability of E6

**DOI:** 10.1371/journal.pone.0112514

**Published:** 2014-11-10

**Authors:** Katia Zanier, Christina Stutz, Susanne Kintscher, Eileen Reinz, Peter Sehr, Julia Bulkescher, Karin Hoppe-Seyler, Gilles Travé, Felix Hoppe-Seyler

**Affiliations:** 1 Institut de Recherche de l'Ecole de Biotechnologie de Strasbourg (IREBS), 67412, Illkirch, France; 2 Molecular Therapy of Virus-Associated Cancers (F065), Program Infection and Cancer, German Cancer Research Center (DKFZ), 69120, Heidelberg, Germany; 3 EMBL-DKFZ Chemical Biology Core Facility, European Molecular Biology Laboratory (EMBL), 69117, Heidelberg, Germany; Bioinformatics Institute, Singapore

## Abstract

The HPV E6 oncoprotein maintains the malignant phenotype of HPV-positive cancer cells and represents an attractive therapeutic target. E6 forms a complex with the cellular E6AP ubiquitin ligase, ultimately leading to p53 degradation. The recently elucidated x-ray structure of a HPV16 E6/E6AP complex showed that HPV16 E6 forms a distinct binding pocket for E6AP. This discovery raises the question whether the E6AP binding pocket is druggable, i. e. whether it provides a docking site for functional E6 inhibitors. To address these issues, we performed a detailed analysis of the HPV16 E6 interactions with two small peptides: (i) E6APpep, corresponding to the E6 binding domain of E6AP, and (ii) pep11**, a peptide that binds to HPV16 E6 and, in contrast to E6APpep, induces apoptosis, specifically in HPV16-positive cancer cells. Surface plasmon resonance, NMR chemical shift perturbation, and mammalian two-hybrid analyses coupled to mutagenesis indicate that E6APpep contacts HPV16 E6 amino acid residues within the E6AP pocket, both *in vitro* and intracellularly. Many of these amino acids were also important for binding to pep11**, suggesting that the binding sites for the two peptides on HPV16 E6 overlap. Yet, few E6 amino acids were differentially involved which may contribute to the higher binding affinity of pep11**. Data from the HPV16 E6/pep11** interaction allowed the rational design of single amino acid exchanges in HPV18 and HPV31 E6 that enabled their binding to pep11**. Taken together, these results suggest that E6 molecular surfaces mediating E6APpep binding can also accommodate pro-apoptotic peptides that belong to different sequence families. As proof of concept, this study provides the first experimental evidence that the E6AP binding pocket is druggable, opening new possibilities for rational, structure-based drug design.

## Introduction

Specific HPV types are closely linked to the development of anogenital and oropharyngeal carcinomas in humans. The best studied cancer entity in this respect is cervical cancer, representing the second most common malignancy in females. Cervical cancers contain in virtually 100% of cases HPV DNA, most prominently HPV type 16 (HPV16) which alone accounts for over 50% of all cervical cancer cases worldwide [1]. HPV-induced malignant cell transformation is primarily linked to the viral E6 and E7 oncogenes [2]–[4]. Their gene products target cellular tumor suppressor proteins for functional inactivation, including p53 and pRb [5], [6].

Notably, the viral E6 and E7 genes are regularly maintained and expressed in cervical cancers. Interference with E6 and/or E7 oncogene expression in HPV-positive cells exerts prominent antitumorigenic effects *in vitro* and *in vivo*, including inhibition of cell proliferation, induction of senescence and activation of apoptosis [7]–[10]. Thus, the malignant phenotype of HPV-positive cancer cells is critically dependent on the expression of the E6 and E7 oncogenes, but is principally reversible by interfering with their activities. In this regard, cervical cancers fulfill the criteria of “oncogene addiction”, i. e. the dependence of tumor cell growth on the activity of one or a few genes [11]. This has important therapeutic implications since it makes the functional inhibition of E6 and E7 a promising therapeutic strategy. Targeting viral factors for therapeutic intervention should, in addition, have the conceptional advantage to enable a specific attack on HPV-positive cells with little or no side effects on undiseased (i. e. virus-negative) cells.

In recent years, the E6 oncoprotein has emerged as a particularly interesting potential therapeutic target. E6 binds to the cellular ubiquitin ligase E6AP (E6-associated protein) and E6/E6AP forms a trimeric complex with p53, resulting in the proteolytic degradation of p53 [6], [12]. It is presumed that the E6/E6AP-induced degradation of the pro-apoptotic p53 protein plays a critical role for maintaining the malignant phenotype of HPV-positive cancer cells. Indeed, we and others have shown that the targeted inhibition of E6 by RNA interference (RNAi) [10], [13], peptide aptamers [7], intracellular antibodies [14], [15] or flavonoids [16] can restore p53 and induce apoptosis in HPV-positive cancer cells. These findings indicate that the anti-apoptotic potential of E6 is important for the survival of HPV-positive cancer cells and suggest that the functional inhibition of E6 represents a promising therapeutic strategy.

As a rational approach, it could be envisioned to generate peptidic E6 inhibitors derived from the binding domain of natural E6 interaction partners, such as E6AP, that may be developed into peptide drugs or serve as lead compounds. Theoretically, such peptides could act as competitive inhibitors of the E6/E6AP interaction, consequently restoring p53 in HPV-positive cancer cells. However, while peptides deduced from the E6-binding domain of E6AP can bind to E6 both *in vitro* and inside cells, they did not affect survival of HPV-positive cancer cells upon intracellular expression [14], [17].

In previous work, we therefore followed an alternative strategy to screen for E6 inhibitors. We identified from a randomized peptide expression library a 15-mer peptide, termed pep11, that specifically binds to the HPV16 E6 protein and does not contain the LXXLL amino acid motif found in several natural E6 interaction partners, such as in E6AP [17]. A solubility-optimized pep11 variant of 19 amino acids in length, termed pep11**, was generated which also specifically binds to HPV16 E6 and, alike pep11, restored p53 and induced apoptosis, selectively in HPV16-positive cells [17]. To the best of our knowledge, pep11** and its variants represent the first bioactive peptides that do not only bind to HPV16 E6 but also can block its anti-apoptotic activity.

Recently, the crystal structure of the 151 amino acid HPV16 E6 protein bound to the E6AP interaction domain (E6APpep) was solved. It revealed that E6 is composed of an N-terminal (E6N) and a C-terminal (E6C) zinc-binding domain which - together with an alpha-helix that connects the two domains - form a distinct hydrophobic binding pocket for E6AP [18]. In view of the central role of the E6/E6AP interaction for HPV-induced carcinogenesis and the potential druggability of HPV16 E6, the structure of the E6/E6AP complex raises important questions. Does the x-ray structure, which employs a solubility-optimized HPV16 E6 mutant [18]), reflect the interaction between E6APpep and wildtype HPV16 E6, at intracellular conditions? Which HPV16 E6 amino acid residues inside, and possibly outside of the pocket, contribute, and to what extent, to E6APpep binding, both *in vitro* and intracellularly? How does E6APpep/E6 binding differ from pep11**/E6 binding, with only the latter interaction inducing apoptosis in HPV16-positive cells [14], [17]? Is there a difference in the ability of the two peptides to restore p53 levels upon binding to E6? Moreover, considering the potential druggability of HPV16 E6, it will be crucial to map the E6 surface interacting with pep11** since it could define a target region for therapeutically useful E6 inhibitors. Thus, what are the E6 residues binding to pep11** and is the E6AP pocket involved in the interaction?

In the present work we investigate the structural determinants of the HPV16 E6/pep11** interaction and compare it to the complex formation between HPV16 E6 and the binding domain of the natural interaction partner E6AP. We show that whereas pep11** docks to the E6AP binding pocket, crucial contributions for specific recognition of pep11** are mainly located within the interdomain linker helix but are also provided by E6 residues adjacent to the pocket. Altogether the data indicate that the pep11** binding surface of E6 defines a potential target region for therapeutically useful E6 inhibitors.

## Results

### Kinetic analyses of HPV16 E6 binding to E6APpep or pep11**

We used surface plasmon resonance (SPR) to investigate the kinetics of the interaction between HPV16 E6 and pep11**, and between HPV16 E6 and E6APpep (an 18-mer peptide corresponding to the E6-binding domain of E6AP [12]). We employed a solubility-optimized mutant of HPV16 E6, named E6 F47R 4C/4S, which harbors the F47R mutation preventing dimerization of the E6N domain and four cysteine to serine substitutions in the E6C domain for suppression of disulfide cross-bridging [19]. Biotinylated peptides comprising either the pep11** sequence or E6APpep were captured on a streptavidin-coated surface. Subsequently, the E6 F47R 4C/4S analyte was injected on these surfaces and the interactions were monitored.

**Figure 1:**
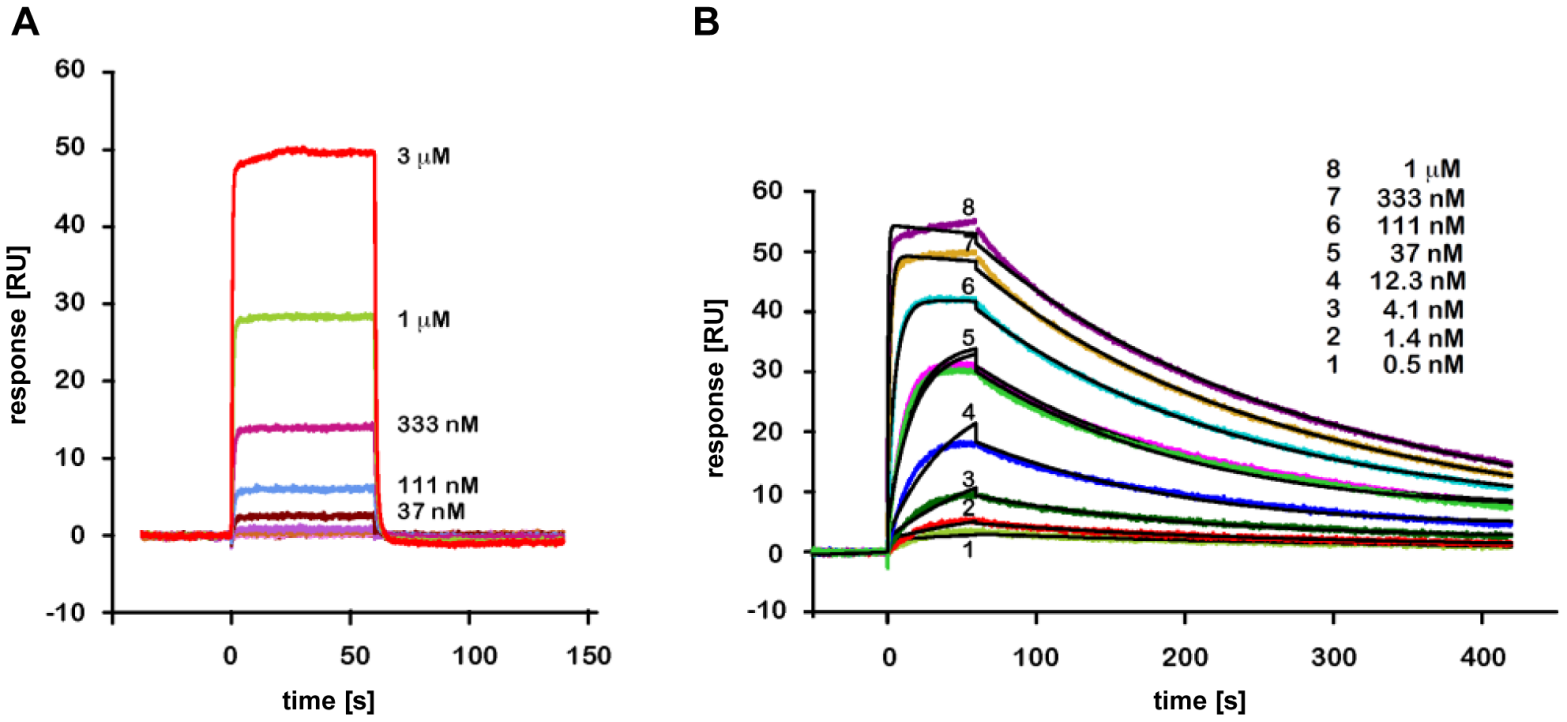
SPR based kinetic analyses of the HPV16 E6/E6APpep and HPV16 E6/pep11** interactions. (*A*) Sensorgrams obtained by injecting purified F47R 4C/4S E6 at the indicated concentrations on a surface capturing E6APpep. RU stands for SPR response units. Color lines represent analyte protein injections at the indicated concentrations. (*B*) Interaction of E6 F47R 4C/4S with surface-captured pep11**. Color and black lines indicate analyte injections and global fits to a 1∶1 binding model, respectively.

The E6/E6APpep interaction is characterized by fast association and dissociation, allowing only equilibrium analysis of the sensorgrams (Figure 1A). An equilibrium dissociation constant K_D_ of 2.3 µM ±0.2 µM was obtained (Figure S1) which is in good agreement with previous SPR experiments that used captured GST-E6APpep fusion as ligand and the HPV16 E6 6C/6S construct as analyte [20]. By contrast, the HPV16 E6/pep11** interaction exhibited a slow dissociation phase (Figure 1B). The fit of the sensorgrams using a simple 1∶1 Langmuir model is not perfect which might indicate a more complex binding model. Nevertheless, the dissociation phase could be fitted separately from the association phase with a k_off_ equal to 0.0051 s^−1^±4.3×10^−6^ s^−1^. Fitting of equilibrium responses of the sensorgrams yielded a K_D_ of 34.0±2.4 nM for the E6/pep11** interaction (Figure S1).

### NMR mapping of the E6APpep and pep11** binding surface on HPV16 E6

**Figure 2:**
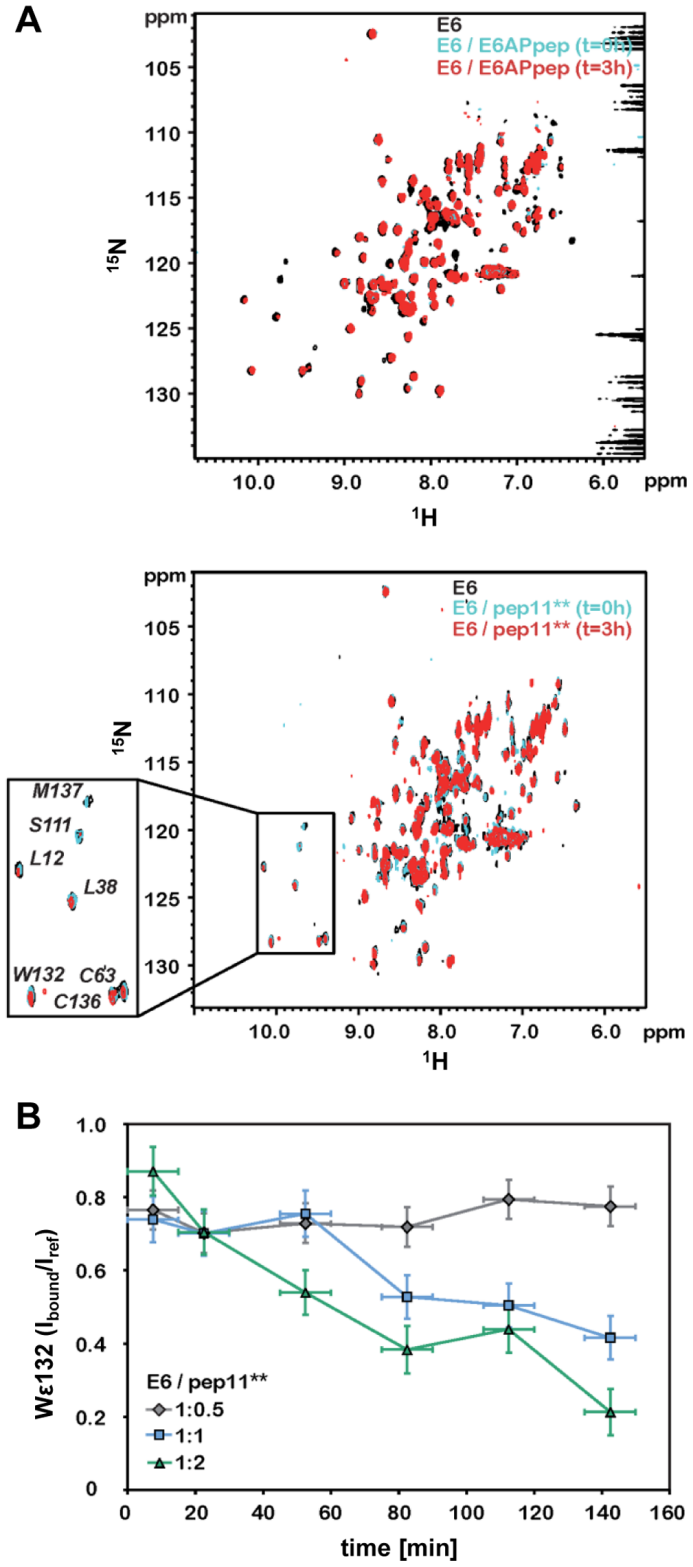
NMR analyses of the HPV16 E6/E6APpep and HPV16 E6/pep11** interactions. (*A*) ^1^H,^15^N SOFAST-HMQC spectra of 100 µM ^15^N E6 F47R 4C/4S samples in the absence (black spectra) and presence of 1∶1 stoichiometric ratios (cyan and red spectra) of unlabeled E6APpep (upper panel) or pep11** (lower panel). Cyan spectra were recorded immediately after peptide addition (t = 0 h), whereas red spectra were recorded after 3 h of incubation in the presence of the peptide. Note that in the case of spectra of E6/pep11** samples some signals have lower intensity in the red spectrum than in the cyan spectrum (see enlarged view of lower panel). This decrease in signal intensity is concomitant with the appearance of a white precipitate of E6 in the NMR tube. (*B*) pep11** induced E6 aggregation monitored by the decrease of the intensity of the W132 indole cross-peak. Intensity changes are expressed as a I_bound_/I_ref_ ratio, where I_bound_ is the cross-peak intensity in the peptide-bound spectra and I_ref_ the cross-peak intensity in the reference (unbound) spectrum. W132 indole intensities were derived from ^1^H,^15^N SOFAST-HMQC spectra recorded at regular time intervals on three different samples containing ^15^N labeled E6 F47R 4C/4S and unlabeled pep11** mixtures with concentration ratios adjusted to 1∶0.5 (gray diamonds), 1∶1 (cyan squares) and 1∶2 (green triangles). E6 concentrations in all three samples were adjusted to 100 µM. X-axis error bars correspond to the duration of the NMR experiment (i.e. error bar: 15 min), whereas y-axis error bars report on the signal-to-noise (S/N) ratio in the reference spectra, which, in this case, is expressed as the inverse of the S/N for sake of clarity (i.e. error bar: ± I_noise_/I_ref_). Estimates of the noise were obtained using the program NMRpipe [32].

Next, we performed NMR chemical shift perturbation (CSP) analyses to investigate the pep11** binding interface on HPV16 E6 and to compare it with the E6 interface specified by the x-ray structure of the HPV16 E6/E6APpep complex [18]. In initial experiments, we observed that, upon addition of unlabeled pep11**, E6 samples displayed formation of solid precipitate paralleled by changes in the NMR spectra (during the first 3–4 hours), whereas E6 samples containing E6APpep remained clear and their spectra did not change over time (Figure 2A). These observations pointed to ligand-induced aggregation in the case of pep11**. Hence, to obtain accurate CSP data, we searched for conditions that minimized contributions from aggregation events occurring during the NMR measurement. First, to record ^1^H-^15^N correlation spectra, we chose the SOFAST-HMQC pulse scheme, which allows reducing the acquisition time compared to classical ^1^H-^15^N HSQC schemes [21]. Then, we explored different sample conditions. Three distinct samples of ^15^N labeled E6 F47R 4C/4S were adjusted to a concentration of 100 µM and mixed with unlabeled pep11** to obtain protein:peptide stoichiometric ratios of 1∶0.5, 1∶1 and 1∶2 respectively. On each sample ^15^N SOFAST-HMQC spectra were recorded immediately after mixing the protein to the peptide (time = 0 spectrum) and then at 30 min time intervals for the following 3−4 h.

Addition of pep11** led to a decrease in the intensity of E6 signals, which became more pronounced as we moved away from the time of mixing the protein to the peptide (Figure 2B). The progress of pep11**-mediated E6 aggregation can be followed from the intensity of the W132 indole cross-peak. This cross-peak was relatively unaffected by peptide binding (with I_bound_/I_free_ values of approximately 0.8 in the time = 0 spectra, see legend of Figure 2B), but sensitive to aggregation (i.e. I_bound_/I_free_ values decreasing with time). From this analysis, it appears that pep11**-mediated E6 aggregation is promoted at higher peptide concentrations, whereas a 1∶1 concentration ratio represents a good compromise between sufficiently strong ligand-induced CSP effects and minimized aggregation.

**Figure 3:**
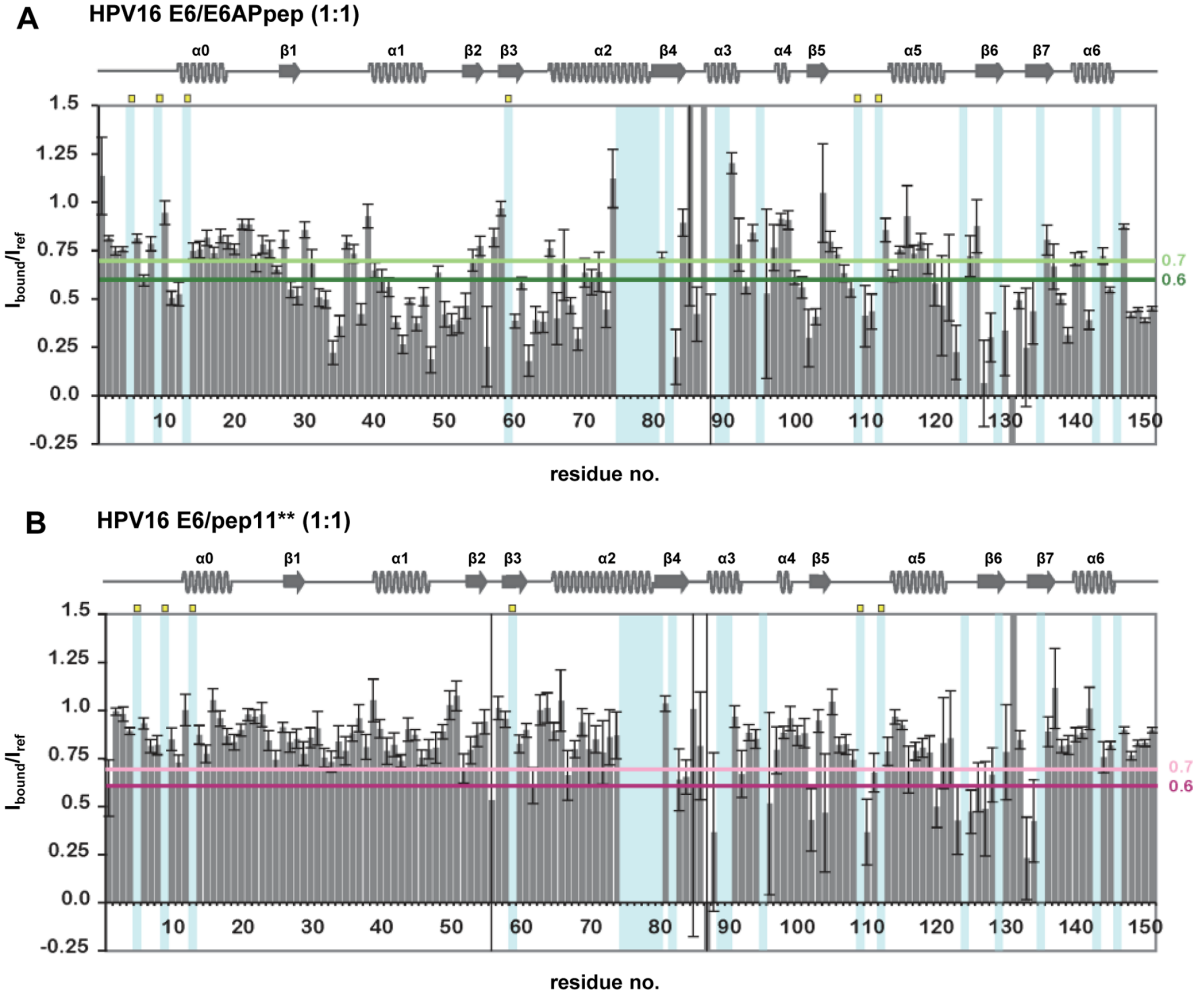
NMR interface analyses of the HPV16 E6/E6APpep and HPV16/pep11** interactions. Indicated are the changes in the intensities of backbone amide cross-peaks of E6 F47R 4C/4S residues upon addition of a 1-fold excess of unlabeled E6APpep (*A*) or pep11** (*B*). I_bound_/I_ref_ thresholds of 0.7 and 0.6 are indicated by colored horizontal lines (light green/dark green and pink/purple for the E6APpep and pep11** interactions, respectively). Error bars (I_noise_/I_ref_ ) report on the signal-to-noise (S/N) ratio in the reference spectra (see legend of Figure 2). Amide cross-peaks with a I_noise_/I_ref_ >0.25 are considered noisy peaks. Cyan shaded areas indicate unassigned residues or prolines (which are additionally highlighted by yellow dots). HPV16 E6 secondary structure elements deduced from the HPV16 E6/E6APpep x-ray structure are indicated above the histograms.

In the final set of experiments, optimized ^15^N SOFAST-HMQC spectra were recorded on E6 F47R 4C/4S samples before and after addition of a 1-fold excess of pep11**. The same experimental conditions were employed to obtain CSP data on the E6/E6APpep interaction. The resulting spectra were analyzed by estimating the intensity changes of the E6_free_ amide cross-peaks upon peptide addition on a per residue basis. Residues with I_bound_/I_free_ ratios below 0.7 were considered to be significantly affected by the interaction with the two peptides, whereas noisy amide cross-peaks were discarded (see legend of Figure 3).

**Figure 4:**
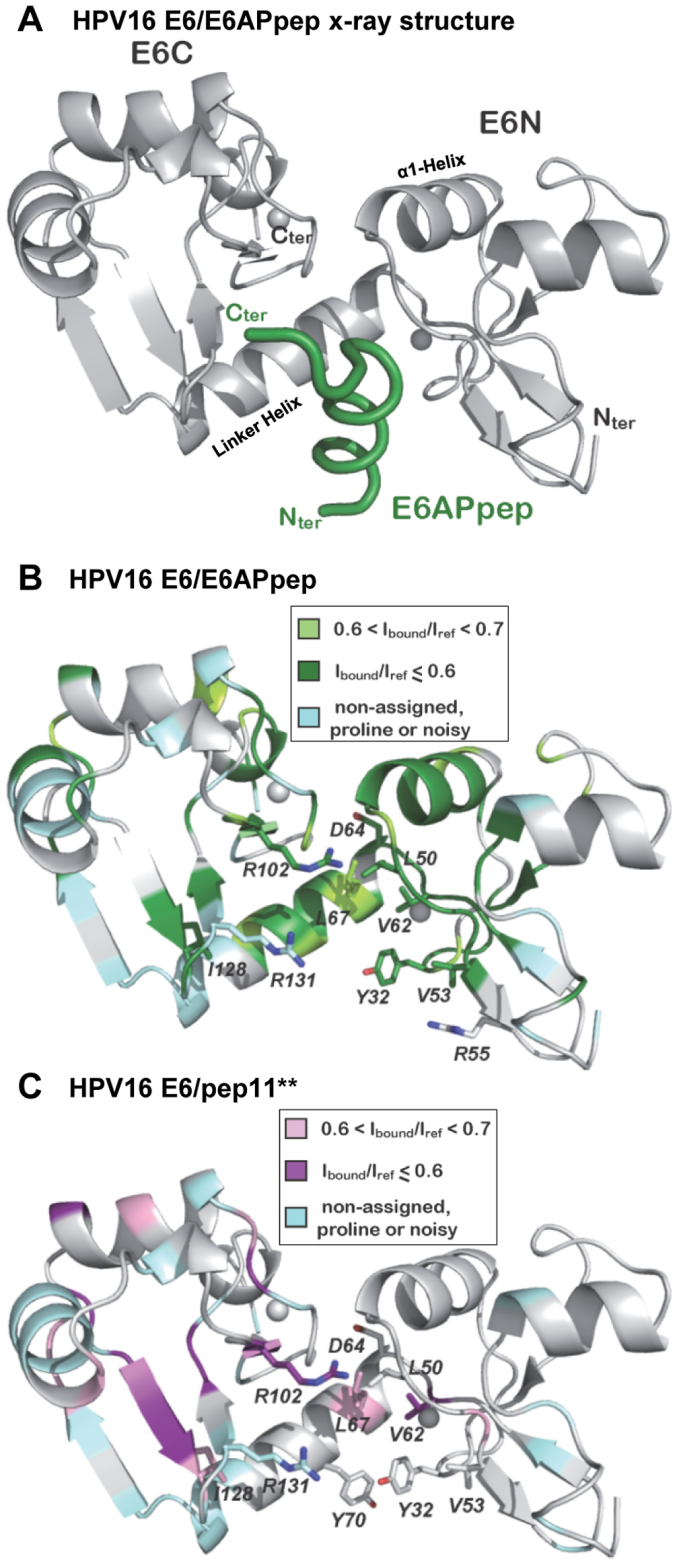
Integrated NMR interface and mutagenesis data illustrated onto the HPV16 E6 3D structure. (*A*) Ribbon presentation of the x-ray structure of HPV16 E6 (gray) bound to E6APpep (green) [18]. (*B*) The HPV16 E6/E6APpep interaction. The color-coding refers to NMR interface mapping data. E6 residues with amide groups having an I_bound_/I_ref_ ratio between 0.7 and 0.6 upon addition of a 1-fold excess of E6APpep (see Figure 3A) are shown in light green, whereas residues with an I_bound_/I_ref_ below 0.6 in dark green. Non-assigned residues, prolines and noisy peaks (with I_noise_/I_ref_ >0.25) are shown in cyan. The side-chains of residues whose mutation results in a reduction of the E6/E6APpep interaction in mammalian two-hybrid assays of 1.5-fold or more are displayed. (*C*) The HPV16 E6/pep11** interaction. As in panel (*B*), color-coding refers to NMR interface mapping data. E6 residues displaying a I_bound_/I_ref_ ratio between 0.7 and 0.6 upon addition pep11** (Figure 3B) are shown in pink and residues with an I_bound_/I_ref_ below 0.6 in purple. Side-chains that when mutated decrease E6/pep11** binding in mammalian two-hybrid assays by 1.5-fold or more (Figure 5C) are displayed.

Overall, it appears that CSP effects are more pronounced for E6 binding to E6APpep than to pep11**, especially in the region of the E6N domain (Figs. 3A–B). Mapping of these CSP data on the structure of the E6/E6AP complex (Figs. 4A–B) reveals a clustering to both the E6AP binding pocket and to other regions, including the E6N dimerisation helix α1 (residues 41–47) and the C-terminal PDZ binding motif (residues 149–151). Perturbation of resonances belonging to regions outside the binding pocket is likely to result from indirect effects, such as conformational changes or differences in the oligomeric states between free and bound E6 forms. From this we deduce that the E6 conformation, with respect to the E6N and E6C orientations and/or the oligomeric state, might be different in the free and E6AP bound forms.

On the other hand, pep11** induced CSP effects on several residues in the E6C domain region and few residues in the E6N domain (Figs. 3B and 4C). Interestingly, most E6 residues affected by pep11** addition also mediate interactions with E6APpep, according to the E6/E6APpep x-ray structure. However, and in contrast to E6APpep, pep11** did not induce CSP effects on the E6N dimerization helix α2 and the C-terminal PDZ binding domain.

Finally, ^15^N-labeled samples of constructs of the isolated E6N and E6C domains (corresponding to residues 1–80 and residues 81–151 respectively [19]) were titrated with a 5-fold excess of pep11**. We observed no changes in the spectra of both domains upon peptide addition and detected perfectly superposable black, cyan and red spectra (Figure S2). This indicates that the interaction with pep11** requires the entire E6 protein, as it is the case for E6APpep binding [20].

### Mutagenesis and intracellular analyses of the HPV16 E6/E6APpep and HPV16 E6/pep11** interactions

Next, we analyzed the intracellular interaction between HPV16 E6 and pep11** or E6APpep, respectively. In order to assess the contribution of specific HPV16 E6 amino acid residues, a panel of mutant HPV16 E6 proteins was created. Individual amino acid exchanges were chosen on the basis of the HPV16 E6 x-ray structure, choosing amino acids present on the surface of HPV16 E6 or protruding into the E6AP binding pocket [18], and by considering their conservation among E6 proteins of different HPV types. A detailed list of the amino acid exchanges and their location on the HPV16 E6 three-dimensional structure is provided in Tab. S1.

Subsequently, wildtype (wt) and mutant (mt) HPV16 E6 proteins were tested for their ability to bind to pep11** or E6APpep intracellularly, by mammalian two-hybrid assay. Specifically, pep11** or E6APpep were expressed as a fusion to the GAL4 DNA binding domain whereas wt and mt HPV16 E6 proteins were expressed as a fusion to the VP16 transcriptional activation domain. Intracellular binding between HPV16 E6 and pep11** or E6APpep was monitored by the stimulation of a co-transfected luciferase reporter plasmid under transcriptional control of GAL4 binding sites [17], [22].

**Figure 5:**
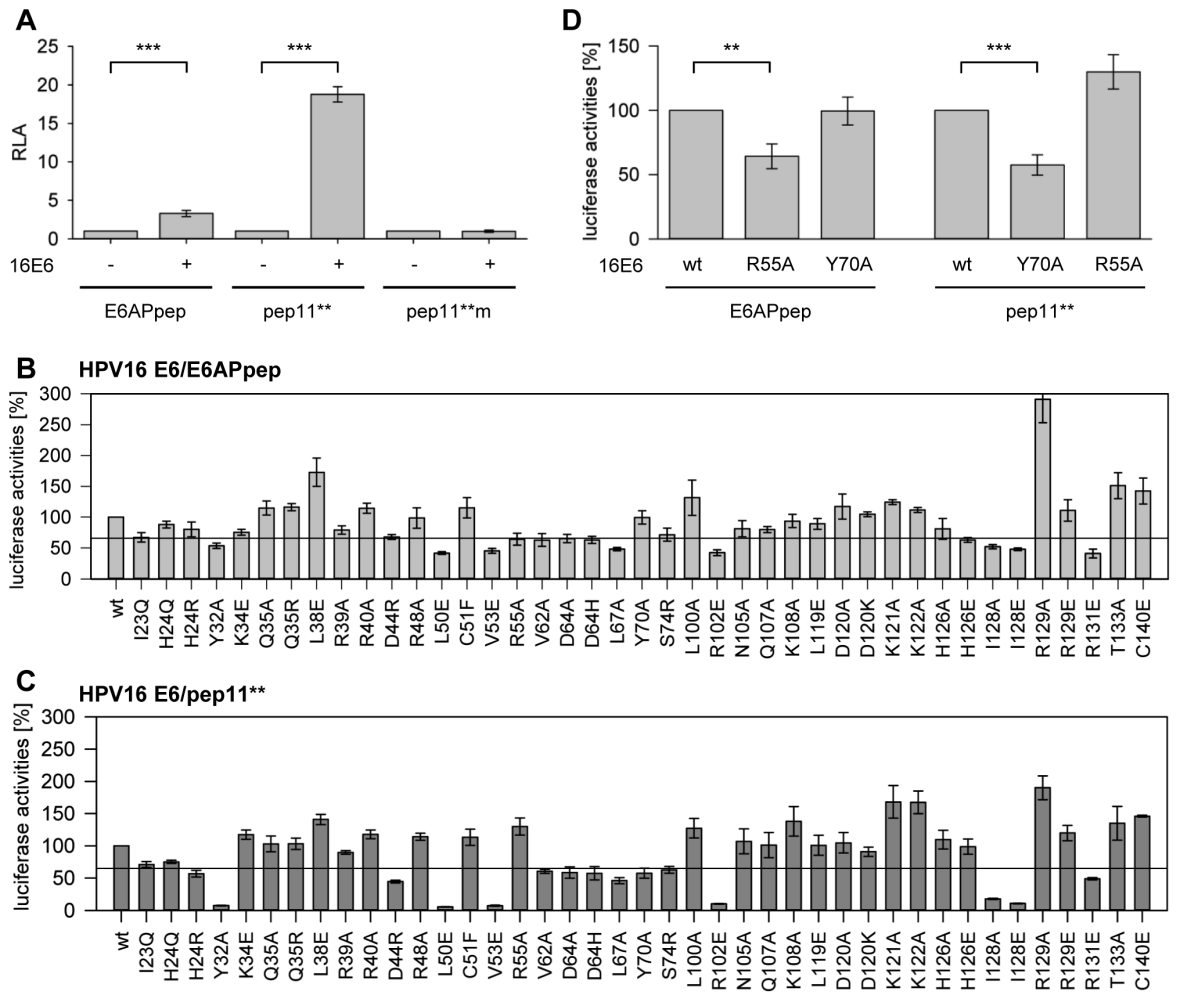
Intracellular analyses of the HPV16 E6/E6APpep and HPV16 E6/pep11** interactions. (*A*) Mammalian two-hybrid analyses in HeLa cells expressing individual peptides, as indicated, linked to GAL4-BD, and wt HPV16 E6 linked to the VP16-AD. pep11**m does not bind to HPV16 E6 and served as a negative control. Shown are relative activities of the co-transfected luciferase reporter plasmid under transcriptional control of GAL4-binding sites above those of control-transfected cells (expressing corresponding peptide-GAL4-BD fusions together with empty control vector pACT; values arbitrarily set at 1.0). Results were obtained from three individual experiments, each performed in duplicates. Standard deviations are indicated. Asterisks above horizontal lines indicate statistically significant differences from pACT-transfected cells, with p-values of ≤0.001 (***). (*B*) and (*C*) Mutational analyses of HPV16 E6 to identify amino acid residues that contribute to E6APpep (*B*) or pep11** (*C*) binding. The luciferase signal for the interaction of wt HPV16 E6 with E6APpep or pep11** was set at 100% (left columns in (*B*) and (*C*), respectively). The horizontal line indicates 1.5-fold inhibition. Individual amino acid exchanges are indicated below each column. Error bars indicate standard deviation values. (*D*) Statistical analysis of the differential binding behaviour of HPV16 E6 mutants 16E6R55A and 16E6Y70A. The values for the interaction between wt HPV16 E6 protein with E6APpep or pep11**, respectively, were set at 100%. Asterisks above the horizontal lines indicate statistically significant reductions of luciferase activities, with p-values of ≤0.01 (**) or ≤0.001 (***). Error bars indicate standard deviation values.

Luciferase activities were increased approximately 3-fold by the HPV16 E6/E6APpep interaction and approximately 19-fold by the HPV16 E6/pep11** interaction (Figure 5A), consistent with the notion that pep11** binds to HPV16 E6 with higher affinity than E6APpep (Figure S1). As negative control, a mutant pep11** variant defective for E6 binding, termed pep11**m [17], did not stimulate luciferase activities (Figure 5A).

Analyses of the binding of a spectrum of different E6 mutants to E6APpep revealed that 10 individual amino acid mutations led to an over 1.5-fold reduction of luciferase activities when compared with the wt HPV16 E6/E6APpep interaction (Figure 5B and Tab. S1). The comparison of these results with the NMR CSP data showed a substantial overlap of the amino acid residues implicated in the E6/E6APpep interaction by both methods (Figure 4B). Among those 10 amino acid residues of HPV16 E6 that were important for intracellular E6APpep binding, 8 establish direct intermolecular contacts with E6AP in the x-ray structure (Figure S3).

Similarly, mutations of distinct residues of HPV16 E6 severely impaired the binding to pep11** (Figure 5C). Several of these residues were also found to interact with pep11** by NMR CSP (Figure 4C), although this time the overlap between the results of the two techniques was not quite as extensive as for the HPV16 E6/E6APpep interaction (Figure 4B). Interestingly, most HPV16 E6 residues which contributed to intracellular E6APpep binding were also important for pep11** binding (Figure 5B–C), corroborating the view that similar amino acid contacts mediate both interactions. However, the mutational analyses also revealed that there exist HPV16 E6 residues that are differentially involved, most prominently R55 and Y70. Specifically, mutation of R55 significantly interfered with the binding to E6APpep but not to pep11** (Figs. 5B–D). *Vice versa*, mutation of Y70 did not markedly affect the E6/E6APpep interaction but significantly reduced the binding of HPV16 E6 to pep11** (Figs. 5B–D).

**Figure 6:**
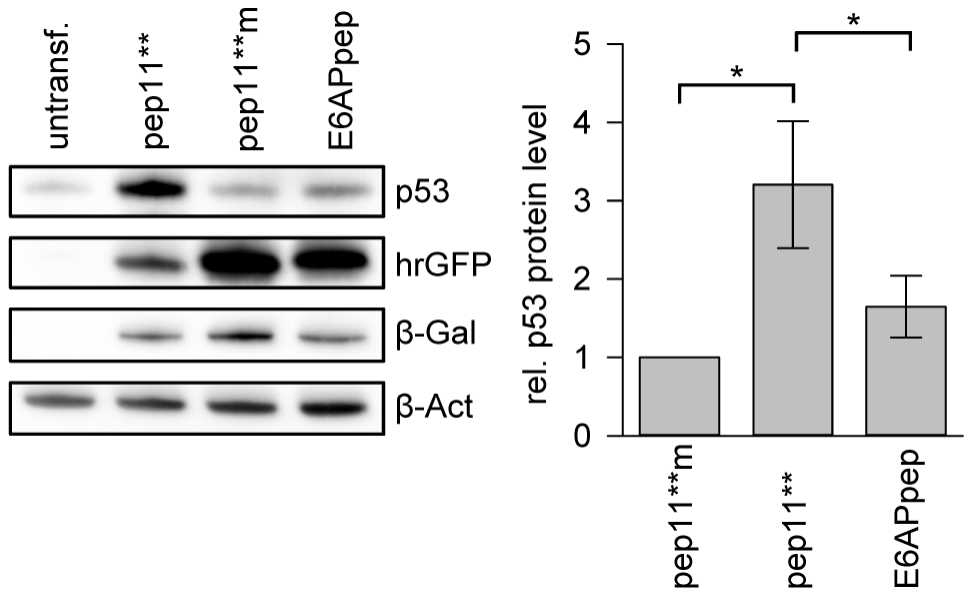
Intracellular p53 protein levels upon pep11** or E6APpep expression in HPV16-positive cells. Left panel: Representative immunoblot analysis following ectopic expression of pep11**, pep11**m, or E6APpep, linked to hrGFP, in HPV16-positive MRI-H186 cells. Expression levels of individual peptide-GFP fusion proteins were measured by employing an hrGFP antibody; β-Gal signals indicate transfection efficiencies; untransf., untransfected cells; β-Act, β-Actin (loading control). Right panel: Densitometric quantification of p53 levels and statistical analysis from 4 independent immunoblot experiments. Amounts of p53 protein in the presence of pep11**m were set at 1.0. Asterisks above the horizontal lines indicate statistically significant differences in p53 amounts, with p-values of ≤0.05 (*). Error bars indicate standard deviation values.

In view of these differences in E6-binding, we investigated whether the peptides show differences in p53 reconstitution. We analyzed the p53 amounts upon intracellular expression of pep11** or E6APpep, respectively, in HPV16-positive MRI-H-186 cells. Peptides were expressed as a fusion with hrGFP for intracellular stabilization and for allowing a comparison of expression levels. As shown in Figure 6. p53 was significantly induced by pep11** compared with untransfected cells or with cells expressing E6-binding defective control peptide pep11**m. Notably, although being expressed at lower levels (at comparable transfection efficiencies), pep11** induced a significantly higher increase of p53 protein amounts than E6APpep (Figure 6). These results indicate that the differences in E6 binding between the two peptides correlate with a more efficient reconstitution of p53 levels by pep11**.

### Single amino acid exchanges can render the E6 proteins of other HPV types competent for pep11** binding

**Figure 7:**
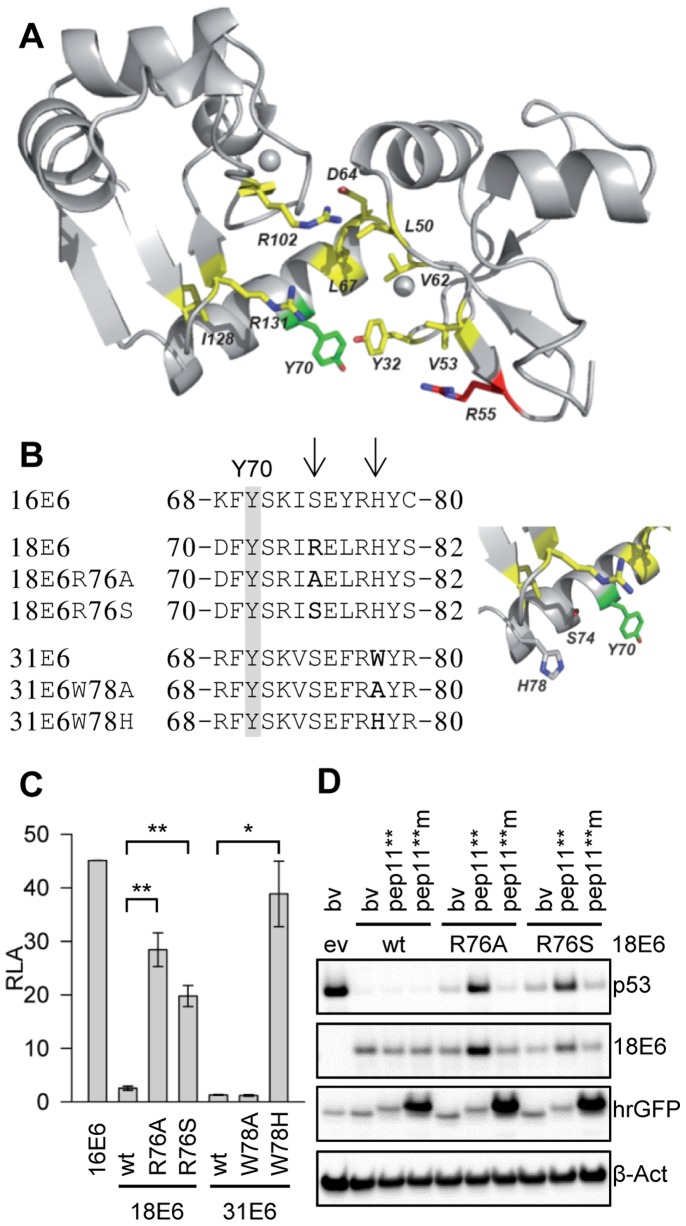
Single amino acid exchanges in HPV18 and HPV31 E6 enable binding to pep11**. (*A*) Amino acid residues involved into the binding to pep11** or E6APpep are illustrated onto the HPV16 E6 3D structure. The side-chains involved in both interactions are shown in yellow; Y70, which is characteristic of the interaction with pep11**, is shown in green, whereas R55, which is characteristic for E6APpep binding, is shown in red. (*B*) *Left panel:* Alignments of interdomain helix linker sequences of HPV16, HPV18 and HPV31 E6 proteins. Positions of amino acid exchanges in mt HPV18 or HPV31 E6 proteins, respectively, are indicated by arrows. HPV16 E6 residue Y70 is highlighted. *Right panel:* 3D view of adjacent side chains of residues Y70, S74 and H78 within the interdomain linker helix of HPV16 E6. (*C*) Mammalian two-hybrid analysis of the interaction between pep11** and wt or mt E6 proteins, as indicated. Shown are relative luciferase activities above those of control-transfected cells (expressing pep11**-GAL4-BD together with empty control vector pACT; arbitrarily set at 1.0). Results were obtained from three individual experiments, each performed in duplicates. Asterisks above horizontal lines indicate statistically significant differences from pACT-transfected cells, with p-values of ≤0.05 (*) or ≤0.01 (**). Standard deviations are indicated. (*D*) Immunoblot analysis of H1299 cells. Cells ectopically express p53 together with wt HPV18 E6 or HPV18 E6 mutants (18E6R76A and 18E6R76S) - and hrGFP-pep11** or hrGFP-pep11**m, as indicated. Expression levels of peptide-GFP fusion proteins were measured by employing an hrGFP antibody. ev, empty expression vector (pcDNA3) devoid of E6 sequences; bv, basic expression vector (pCEP4-hrGFP [17]) devoid of pep11** or pep11**m sequences; β-Act, β-Actin (loading control).

The positioning on the 3D structure of the amino acid residues whose mutations affected intracellular binding of HPV16 E6 to E6APpep or to pep11**, respectively, is depicted in Figure 7A. Notably, E6APpep can bind to both HPV16 and HPV18 E6 whereas pep11** binds HPV16 E6 but only very weakly interacts, if at all, with HPV18 E6 [17]. In order to identify possible structural determinants for this discrepancy, we compared the amino acid sequences of the contact region in HPV16 E6 with the corresponding domain of HPV18 E6. HPV16 E6 amino acid Y70 - which differentially affected pep11** and E6APpep binding (Figs. 5B–D) - has a correlate in residue Y72 in HPV18 E6 (Figure 7B). However, in the vicinity of Y72, HPV18 E6 contains an arginine at position 76 (R76) that corresponds to S74 in HPV16 E6 (Figure 7B), a residue which was found to be important for pep11** binding by HPV16 E6 (Figure 5C). We hypothesized that in HPV18 E6 the larger R76 residue may protrude into a potential pep11** binding pocket and thereby could interfere with the interaction between pep11** and residues in the E6AP binding pocket. Alternatively, a serine residue at this position, as present in HPV16 E6, could directly contribute to pep11** binding by providing polar interactions. To test this issue, we created two HPV18 E6 mutants, introducing either an alanine (18E6R76A) or a serine (18E6R76S) at amino acid position 76 (Figure 7B).

Co-expression of HPV18 E6 with pep11** led to only a very modest activation (about two-fold) of the luciferase reporter in mammalian two-hybrid assays (Figure 7C), in line with previous data showing that pep11** binds only very weakly to HPV18 E6, if at all [17]. Remarkably, however, a single amino acid change of HPV18 E6 residue R76 to either S76 (18E6R76S) or A76 (18E6R76A) strongly increased the intracellular binding capacity of HPV18 E6 to pep11** (Figure 7C). These data show that the introduction of a less bulky amino acid at this position can render HPV18 E6 competent for pep11** binding.

Similar analyses were performed for the interaction between pep11** and HPV31 E6. HPV16 E6 and HPV31 E6 share many amino acids that were found to be important for pep11** binding, including S74 at corresponding positions (Figure 7B). Yet, HPV31 E6 does not detectably interact with pep11** (Figure 7C). Inspection of the amino acid sequences revealed that HPV31 E6 contains a tryptophan at position 78 whereas HPV16 E6 contains a histidine at the corresponding site (Figure 7B). We replaced HPV31 E6 W78 by either histidine (31E6W78H) or alanine (31E6W78A). Notably, 31E6W78H, but not 31E6W78A, rendered HPV31 E6 competent for pep11** binding (Figure 7C). These findings indicate that the presence of a histidine at this site - as found in the corresponding region of HPV16 E6 - is critical for binding to pep11**.

Finally, we examined whether mutations enabling efficient pep11** binding renders E6 sensitive for inhibition by pep11**. To this end, we co-expressed p53, wt or mt HPV18 E6 proteins, and pep11** or pep11**m in H1299 cells (which do not contain endogenous p53). HPV18 E6 almost completely depleted the cells of co-expressed p53 protein (Figure 7D), in line with its ability to mediate p53 degradation. Notably, p53 depletion by wt HPV 18 E6 was not affected by co-expression of pep11**. In contrast, co-expression of pep11** with the pep11**-binding competent 18E6R76A or 18E6R76S mutants resulted in two effects. First, pep11**, but not E6-binding defective pep11**m, induced an increase of 18E6R76A and 18E6R76S levels. This finding is reminiscent of a report indicating that molecules binding to E6, such as E6AP, can result in E6 stabilization [23]. Second, at the functional level, pep11** was able to counteract p53 depletion by the pep11**-binding competent 18E6R76A and 18E6R76S mutants (Figure 7D).

Taken together, these results show that the structural and functional data obtained for the E6/pep11** interaction allows educated guesses to create single amino acid exchanges that render E6 proteins from other HPV types competent for pep11** binding. This provides further strong experimental support for the correctness of the proposed interface of the HPV16 E6/pep11** interaction.

## Discussion

The HPV E6 oncoprotein is considered to represent an attractive therapeutic target to combat HPV-linked neoplasias and, possibly, HPV-induced preneoplasias. The recent observation by X ray analysis that E6 forms *in vitro* a distinct binding pocket for E6AP shed new light on this interaction which is considered to be crucial for HPV-induced carcinogenesis. It also provides a basis for a detailed experimental analysis to understand the molecular nature of the E6/E6AP recognition, both *in vitro* and at the intracellular level. Furthermore, it raises the question whether E6 may be druggable in that the E6AP binding pocket may provide a docking site for functional E6 inhibitors.

To address these issues, we here analyzed the interaction between HPV16 E6 and two peptides: E6APpep, an 18-mer peptide comprising the binding domain of the natural interaction partner E6AP [12], and pep11**, a pro-apoptototic, HPV16 E6-binding 19-mer peptide derived from a randomized peptide expression library [17]. Our results indicate that (i) E6APpep binds – both *in vitro* and intracellularly – to amino acid residues that form a E6AP binding pocket according to x-ray data [18], (ii) several of those amino acids appear to be involved in both the HPV16 E6/E6AP and HPV16 E6/pep11** interactions, suggesting that the two peptides contact a similar region of HPV16 E6, (iii) yet, specific differences were detected for distinct amino acid residues, (iv) the different binding modes of E6APpep and pep11** were linked to a higher reconstitution of p53 by pep11** in HPV16-positive cells, and (v) the experimental data obtained for the HPV16 E6/pep11** interaction enabled the rational design of single amino acid exchanges that rendered the E6 proteins of other HPV types competent for pep11** binding.

We have previously shown that the intracellular expression of pep11**, but not of E6APpep, led to the induction of apoptosis, specifically in HPV16-positive tumor cells [17]. We here show that these different phenotypic effects are associated with different E6-binding modes. Specifically, SPR analyses revealed that E6APpep dissociates much faster from HPV16 E6 than pep11** and that pep11** binds to HPV16 E6 with higher affinity than E6APpep, consistent with results from previous fluorescence polarization analyses [17] and from intracellular binding studies, as discussed below.

In order to elucidate possible structural determinants for the different binding affinities and kinetics, we attempted to identify HPV16 E6 residues involved in the binding to E6APpep and pep11**, using NMR CSP. These experiments were based on the analysis of backbone amide groups, since the 2D ^1^H-^15^N correlation spectrum of the monomeric E6 construct used is relatively well dispersed and could be assigned to 92% of completeness [19]. After optimization of the experimental setup, we were able to record NMR spectra of E6 bound to pep11** or E6APpep at conditions of minimal E6 aggregation. Addition of both peptides caused line-broadening of E6 amide cross-peaks. Whereas in the case of E6APpep this can be explained by the affinity of the interaction with E6 (K_D_ = 2.3 µM), which is consistent with an intermediate exchange regime, line broadening is surprising for pep11** peptide. The higher affinity of pep11** (K_D_ = 34 nM) rather suggests a slow exchange regime, which would normally produce a second set of resonances in the spectra belonging to the E6 bound form. Therefore we deduce that the line-broadening observed upon addition of pep11** probably derives from multiple conformations of the peptide in the E6 binding pocket.

A limitation of the CSP analysis based on amide resonances is that ^15^N chemical shifts are sensitive to structural changes [24]. This is particularly evident in the case of the E6APpep interaction, which, differently from pep11**, induces substantial CSP effects on residues outside the E6AP binding pocket, including the α1 self-association helix in E6N and the PDZ binding motif. A second limitation of this approach is that it downplays hydrophobic contributions, since amide chemical shifts are less influenced by this type of interactions [24]. Indeed this might be the reason why only few significant CSP effects are observed for the pep11** interaction in the E6N domain region, which contributes most hydrophobic amino acid residues to the E6AP binding pocket [18]. On the other hand the implementation of side-chain methyl NMR spectroscopy, which has been recently proposed as a valuable tool to study protein-ligand interfaces [24], was hindered by severe line broadening that prevents observation and assignment of several side-chain resonances in full-length E6 spectra [19]. The comparative CSP analysis suggested that a common set of amino acid residues are involved in establishing the intermolecular contacts for both the HPV16 E6/pep11** and the HPV16 E6/E6APpep interactions whereas the overall conformations of these complexes are likely to differ.

Given the limitations described above, it was important to complement the NMR studies by mutagenesis coupled to binding assays. We chose the mammalian two-hybrid system for several reasons. First, the assay detects the intracellular binding of two interaction partners and therefore is informative whether *in vitro* binding analyses (e.g. x-ray structure, NMR) correctly reflect the interactions inside mammalian cells. Second, the assay enables binding analyses of wt HPV16 E6 whereas *in vitro* binding studies of E6 usually require the use of solubility-optimized mt E6 proteins, such as E6 F47R 4C/4S [18]. Third, the assay allows to perform straightforward mutational analyses to identify those E6 amino acid residues that are critical for the interaction with pep11** or E6APpep.

In line with the *in vitro* data, the results from mammalian two-hybrid analyses indicate that pep11** binds stronger to HPV16 E6 than E6APpep. In order to identify specific HPV16 E6 amino acids that are involved in the interaction with the two peptides, we generated a broad spectrum of mt E6 proteins by introducing individual amino acid exchanges within the E6AP binding pocket or on the E6 surface, either close to or distant from the pocket. We found that mutations of amino acid residues outside of the E6AP binding pocket did not inhibit E6APpep binding with the exception of a borderline inhibitory effect of the HPV16 E6 H126E mutation. In contrast, mutations of 10 of the 15 amino acid residues within the E6AP binding pocket interfered with the binding to E6APpep. Interestingly, those amino acids that - according to the x-ray structure - are key residues for the HPV16 E6/E6AP interaction (R102 and R131) also showed particular high detrimental effects in mammalian two-hybrid analyses upon mutation. The amino acids that affect intracellular E6APpep binding upon mutation are mapped on the HPV16 E6 x-ray structure in Figure S3. Taken together, these results are in full agreement with the x-ray structure of the HPV16 E6/E6APpep complex [18] and indicate that the E6AP pocket also mediates the interaction between E6APpep and wt HPV16 E6 inside mammalian cells.

In addition, we found that many HPV16 E6 residues that play a role for E6APpep binding were also involved in the intracellular interaction with pep11**. Consistent with the CSP results, this further corroborates the view that similar amino acid residues are important for both interactions. However, we also identified distinct residues that were differentially involved. Specifically, R55 was more important for the interaction of HPV16 E6 with E6APpep whereas Y70, located within the interdomain linker helix, was more important for the interaction with pep11**. These differences may contribute to the higher affinity observed for the pep11**/HPV16 E6 interaction and to the more efficient p53 reconstitution upon pep11** expression in HPV16-positive cells.

Given that our concept of the HPV16 E6/pep11** interface is correct, we reasoned that it may allow us to render E6 proteins from other HPV types competent for pep11** binding. The mutagenesis data indicated that HPV16 E6 amino acid residue Y70 is a key residue for pep11** binding. Yet, the structurally related HPV18 E6 protein also possesses a tyrosine at the corresponding position (Y72) but is only very weakly bound by pep11**, if at all. Inspection of the HPV18 E6 residues nearby Y72 identified an arginine at position 76 (R76), which corresponds to S74 in HPV16 E6. Replacing HPV18 E6 R76 by smaller amino acids, such as serine or alanine, allowed efficient binding to pep11**. This provides evidence that the R76 residue within wildtype HPV18 E6 prevents pep11** binding by steric hindrance. Similarly, HPV31 E6 could also be rendered competent for pep11** binding by a single amino acid exchange. Here, replacement of W78 by histidine (the amino acid found in the corresponding region of HPV16 E6), but not by alanine, allowed efficient binding of HPV31 E6 to pep11**. As examined for HPV18 E6, the structural modifications enabling pep11** binding were mirrored by functional consequences. Specifically, the ability to deplete cells for p53 was counteracted by pep11** only for pep11**binding-competent mt HPV18 E6 proteins, but not for wt HPV18 E6 protein.

Taken together, these results show that distinct single amino acid exchanges - rationally designed on the basis of the HPV16 E6/pep11** interface data - can render E6 proteins from other HPV types competent for pep11** binding. This strongly supports the validity of our model of the E6/pep11** interface, as deduced from NMR and mammalian two-hybrid analyses. It further highlights the role of residues of the interdomain linker helix in modulating the recognition of peptide ligands targeting the E6AP binding pocket.

Moreover, the observation that the pro-apoptotic pep11** peptide [17] contacts HPV16 E6 very similarly as E6APpep, but with higher affinity, indicates that the anti-apoptotic activity of E6 in HPV-positive tumor cells is amenable to inhibition by therapeutic molecules binding close to, or within, the E6AP binding pocket. This data provides the first experimental evidence that the E6AP binding pocket renders the E6 protein druggable by serving as a docking site for inhibitory ligands, such as pep11**, that exhibit no obvious sequence homologies to the E6AP binding domain. Moreover, the detection of a druggable binding pocket is a pre-requisite for structure-based drug design [25], [26]. For this purpose, it is also important to explore the pocket space for amino acid contacts that favor binding with high affinity [25], [26]. The comparative analysis of E6APpep/E6 and pep11**/E6 binding identified amino acids within the E6AP pocket that were preferentially or selectively targeted by the high affinity pep11** interaction. These residues thus may provide particularly interesting contact points to be considered for the design of therapeutically useful compounds targeting the E6AP binding pocket, such as peptide drugs or peptide mimetics which increasingly find their way into the clinic [27], [28].

## Materials and Methods

### Protein purification

HPV16 E6 DNA constructs for the full-length protein (E6 F47R 4C/4S) and the isolated E6N and E6C domains (i.e. E6N and E6C F47R 4C/4S) have been previously described [19], [29]. All E6 protein constructs were expressed as fusions to the maltose binding protein (MBP) in *E. Coli* BL21 DE3 cells grown in LB or M9 minimal media, supplemented with ^15^NH_4_Cl to allow for ^15^N isotope labeling. The purification protocol [30] consisted of a first purification step using amylose-affinity chromatography, an overnight ultracentrifugation run to eliminate soluble aggregates, a TEV protease cleavage to separate E6 from the MBP tag, and a final gel filtration chromatography step.

### Peptide synthesis

Unmodified pep11** (sequence: KEKEEYNSNCSCIACIGLI) and biotinylated pep11** peptides were chemically synthesized by Peptide Speciality Laboratory (Heidelberg, Germany). Unmodified E6APpep (amino acid residues 398–415 of human E6AP, sequence: IPESSELTLQELLGEERR) and biotinylated E6APpep were chemically synthesized by the Peptide Synthesis Core Facility of the German Cancer Research Center, Heidelberg, Germany. Biotinylated peptides harbored an N-terminal biotin residue, separated from the peptide sequence by four ethylene glycol blocks (PEG3 linker, Novabiochem, Merck, Darmstadt, Germany).

### Surface plasmon resonance

Binding studies were performed on a Biacore T100 system (Biacore, GE Healthcare). Biotinylated peptides were immobilized to a SA (streptavidin)-chip and purified E6 F47R 4C/4S was injected as analyte. Flow cell 1 with no immobilized ligand served as reference. Ligands and analyte were diluted in 20 mM sodium phosphate (pH 6.8), 200 mM NaCl, 2 mM DTT and 0.05% Tween 20. The biotinylated peptides were injected at 30 nM aiming for an immobilization level of not more than 100 RU. Three-fold serial dilutions of E6 were injected for 60 sec at a flow rate of 30 µl/min, followed by 360 sec dissociation phase. After each cycle the surface was regenerated by injecting 50 mM NaOH, 1M NaCl, and 10 mM DTT. Sensorgrams were evaluated with Biacore T100 Evaluation Software, version 2.0.1.

### NMR spectroscopy

^1^H,^15^N-HSQC and ^1^H,^15^N-SOFAST-HMQC [21] spectra were recorded on 600 and 700 MHz spectrometers equipped with cryoprobes (Bruker, Billerica, MA, USA) at 296 K. ^15^N labeled E6 samples were adjusted to a concentration of 100 µM in NMR buffer (20 mM sodium phosphate (pH 6.8), 200 mM NaCl, 2 mM DTT). E6/E6APpep or E6/pep11** samples were prepared by adding small aliquots of a concentrated stock solution of unlabeled peptide (4 mM peptide in NMR buffer) to the labeled E6 samples to match the stoichiometric ratios indicated in the text. The datasets used for the final analysis presented in Figs. 3 and 4 were obtained by recording ^1^H,^15^N-SOFAST-HMQC experiments on protein:peptide mixtures adjusted at 1∶1 stoichiometric ratios on a 700 MHz NMR spectrometer. The total duration of the final ^1^H,^15^N-SOFAST-HMQC experiments was 25 min (for 32 scans, 140 points in the indirect dimension, a relaxation delay of 0.25 sec and other parameters as described [21]). Spectral changes were followed by monitoring amide cross-peak intensities. Backbone resonance assignments for the E6 F47R 4C/4S construct were obtained as described [19]. Spectra were analyzed by computer aided resonance assignment [31].

### Cell culture, plasmids and transfections

HeLa (HPV18-positive) and MRI-H-186 (HPV16-positive) cervical carcinoma cells, and H1299 lung cancer cells were grown in DMEM (pH 7.2), supplemented with 10% fetal bovine serum (Gibco Life Technologies, Carlsbad, CA, USA), 2 mM L-glutamine, 100 U/ml penicillin and 100 µg/ml streptomycin (Sigma-Aldrich, Saint Lois, MO). Plasmids were transfected by calcium phosphate co-precipitation as described [17]. For mammalian two-hybrid analyses, peptides E6APpep, pep11**, or pep11**m (sequence: KEKEEYNSNSSSIASIGLI) were expressed as a fusion to the GAL4 DNA binding domain (GAL4-BD) from vector pBIND. Full-length wildtype or mutant E6 proteins were expressed as a fusion to the VP16 transcriptional activation domain (VP16-AD) from vector pACT [17]. E6 constructs used in Figure 7C contain an additional flag-tag between E6 and the VP16-AD. Mutant derivatives of HPV16, HPV18 and HPV31 E6, as specified in the text, were generated by site directed mutagenesis [17] and verified by DNA sequencing. For ectopic expression of pep11**, pep11**m, and E6APpep for immunoblot analyses, the peptide sequences were expressed as a fusion to humanized recombinant green fluorescent protein (hrGFP; Stratagene, Heidelberg, Germany) from vector pCEP4 (Invitrogen, Karlsruhe, Germany), as described [17]. To each transfection, equal amounts of β-Galactosidase expressing plasmid pCMV-Gal [10] was added to allow comparisons of transfection efficiencies. p53 reconstitution experiments (Figure 7D) were performed as described [17].

### Mammalian two-hybrid assays

The CheckMate system (Promega, Madison, WI, USA) was used to investigate the binding of pep11** or E6APpep to E6, in mammalian cells. Both pBIND and pACT fusion constructs were transfected into HeLa, along with the GAL4-responsive luciferase reporter construct pG5luc and internal standard pCMV-Gal. Two days after transfection, cells were harvested. Luciferase activities were determined as duplicates in at least three independent experiments using a Lucy 1 microplate luminometer (Anthos, Krefeld, Germany) and normalized for pCMV-Gal activities.

### Immunoblot analyses

Cellular protein was extracted in RIPA buffer (10 mM Tris-HCl pH 7.5, 150 mM NaCl, 1 mM EDTA, 1% NP40, 0.5% sodium deoxycholate, 0.1% SDS) two days after transfection. Approximately 15 µg of protein was separated by a NuPage 4–12% Bis-Tris protein gel (Thermo Fisher Scientific, Waltham, MA, USA) and subsequently electrotransferred to an Immobilon-P membrane (Millipore, Bedford, MA, USA) using the Trans-Blot Semi-Dry Transfer Cell (Bio-Rad, München, Germany). Membranes were blocked with 5% skim milk powder (Saliter, Obergrünzburg, Germany) and 1% bovine serum albumin (BSA, Sigma-Aldrich) in PBS-T (PBS 0.2%, Tween-20) for 1 h at room temperature. Membranes were incubated with primary antibodies overnight at 4°C in PBS-T/5% skim milk powder/1% BSA, followed by incubation with the corresponding HRP-conjugated secondary antibody for 1 h at room temperature. Proteins were visualized using ECL Prime Western Blotting Detection Reagent (GE Healthcare, Buckinghamshire, UK). Images were monitored using Fusion SL Gel Detection System (Vilber Lourmat, Marne-la-Vallée, France). Band densities were determined by Bio1D image analysis software (Vilber Lourmat), relative to the respective loading controls. The following primary antibodies were used: anti-p53 antibody DO-1 (Santa Cruz Biotechnology, Dallas, TX, USA), anti-HPV18E6 antibody clone 399 (Arbor Vita Corporation, Sunnyvale, CA, USA), anti-β-galactosidase antibody Z3783 (Promega), anti-hr-GFP antibody [17], and anti-β-actin antibody AC-74 (Sigma).

### Statistical analyses

Statistical significance of differences in measured variables between controls and treated samples was evaluated by a two-sided paired t-test using the Sigma Plot software (Systat Software Inc., San Jose, CA). p-values of ≤0.05 (*), ≤0.01 (**), or ≤0.001 (***) were considered statistically significant.

## Supporting Information

Figure S1 SPR analyses of the HPV16 E6/E6APpep and HPV16 E6/pep11** interactions. (A) Equilibrium responses (Req) of the HPV16 E6/E6APpep interaction plotted as a function of E6 concentrations and fitted to a 1∶1 binding model. (B) *Upper left panel:* Equilibrium responses for the E6/pep11** interaction plotted as a function of E6 concentration and fitted to a 1∶1 binding model. *Upper right panel:* Fitting of the dissociation phase of the E6/pep11** sensorgrams. *Lower panel:* Residual values for dissociation phase fits of the E6/pep11** interaction.
(PDF)

Figure S2 Lack of interaction between pep11** and the isolated HPV16 E6N and E6C zinc binding domains. ^1^H,^15^N SOFAST-HMQC spectra of 100 µM ^15^N labeled samples of the F47R E6N domain (residues 1–80) and E6C 4C/4S domain (residues 81–151) in the absence (black spectrum) and presence of a 5-fold excess of unlabeled pep11** (cyan and red spectra). The cyan spectrum was recorded immediately after peptide addition (t = 0 h), whereas the red spectrum was recorded after 3 h of incubation in the presence of the peptide.
(PDF)

Figure S3 **Projection of the mammalian two-hybrid data for the E6APpep interaction on the E6 structure.** Cyan: residues identified by x-ray analysis to form the interaction domain for E6APpep [12]. Side chains of residues that are required for E6APpep binding, as revealed by mutational analyses in mammalian two-hybrid assays, are indicated.
(PDF)

Table S1 Mutations reducing intracellular binding of HPV16 E6 to E6APpep or pep11**. The location of HPV16 E6 amino acid residues in the HPV16 E6 structure and their involvement in E6APpep binding according to x-ray data [12] are listed. For mammalian two-hybrid analyses, luciferase values for the interaction of wildtype HPV16 E6 with E6APpep or pep11**, respectively, were set at 100%. Shown are relative luciferase activities (RLA) in percent upon mutation of the indicated amino acid residues. Mutations resulting in an over 1.5-fold reduction are highlighted in bold. Standard deviations are indicated. * : within the E6N self-association interface [19].
(DOCX)
