# Supplementary figures and images for: The E6AP Binding Pocket of the HPV16 E6 Oncoprotein Provides a Docking Site for a Small Inhibitory Peptide Unrelated to E6AP, Indicating Druggability of E6
Source: PLoS One. 2014 Nov 10;9(11):e112514. doi: 10.1371/journal.pone.0112514 (PMC4226571; doi:10.1371/journal.pone.0112514)

Figure S1.

A

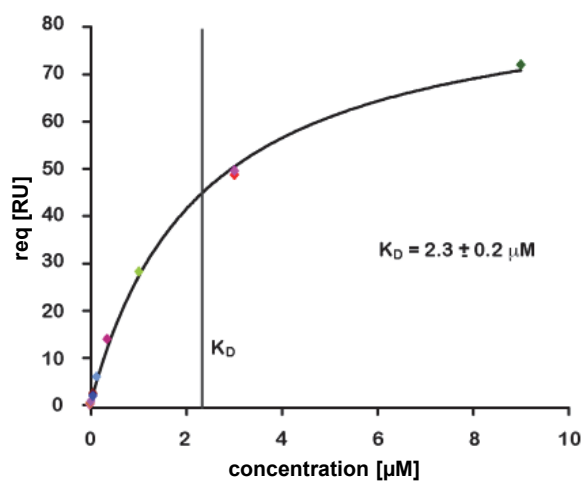

B

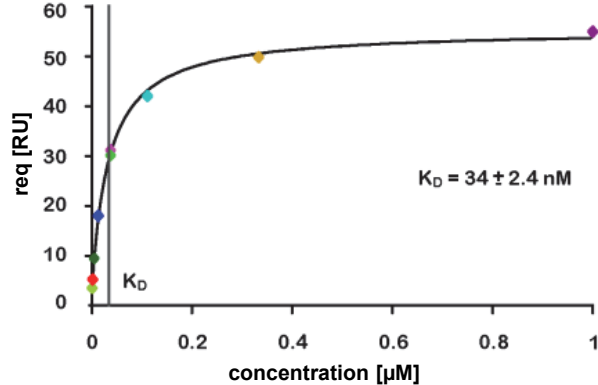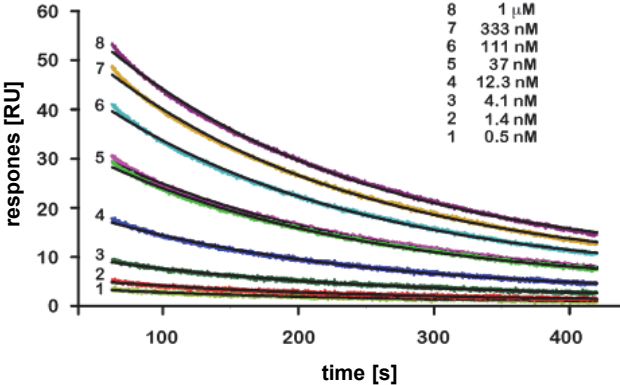

Residuals

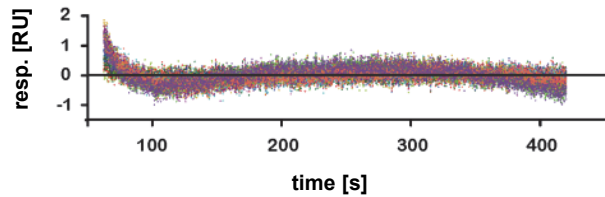

Supplement: Figure S1 — SPR analyses of the HPV16 E6/E6APpep and HPV16 E6/pep11** interactions. (A) Equilibrium responses (Req) of the HPV16 E6/E6APpep interaction plotted as a function of E6 concentrations and fitted to a 1∶1 binding model. (B) Upper left panel: Equilibrium responses for the E6/pep11** interaction plotted as a function of E6 concentration and fitted to a 1∶1 binding model. Upper right panel: Fitting of the dissociation phase of the E6/pep11** sensorgrams. Lower panel: Residual values for dissociation phase fits of the E6/pep11** interaction. (PDF) [file pone.0112514.s001.pdf]

Figure S2.

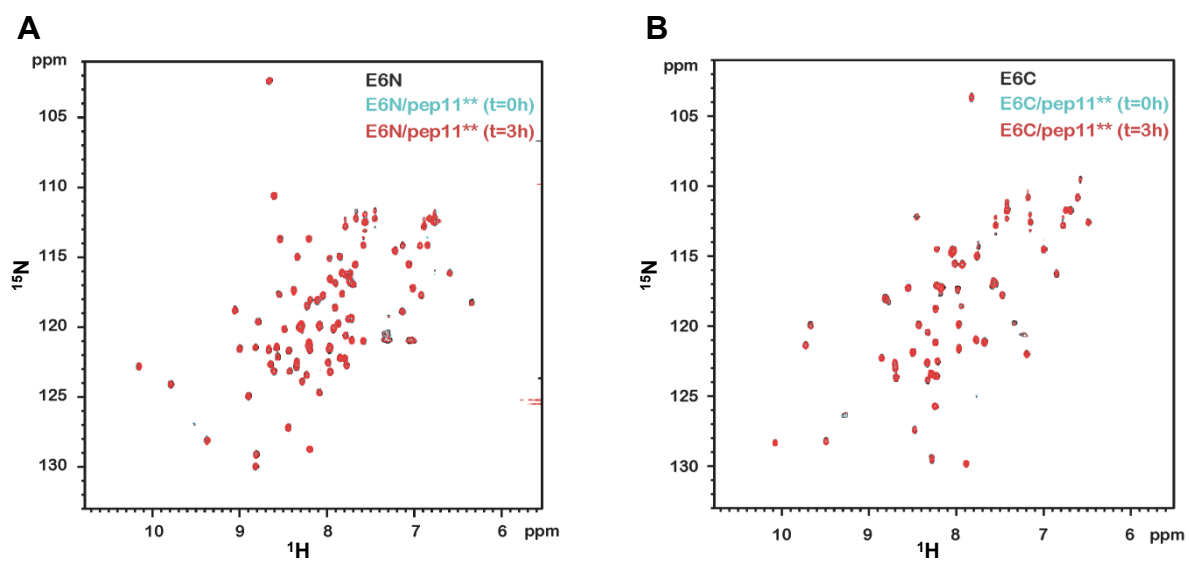

Supplement: Figure S2 — Lack of interaction between pep11** and the isolated HPV16 E6N and E6C zinc binding domains. 1H,15N SOFAST-HMQC spectra of 100 µM 15N labeled samples of the F47R E6N domain (residues 1–80) and E6C 4C/4S domain (residues 81–151) in the absence (black spectrum) and presence of a 5-fold excess of unlabeled pep11** (cyan and red spectra). The cyan spectrum was recorded immediately after peptide addition (t = 0 h), whereas the red spectrum was recorded after 3 h of incubation in the presence of the peptide. (PDF) [file pone.0112514.s002.pdf]

**Figure S3.**

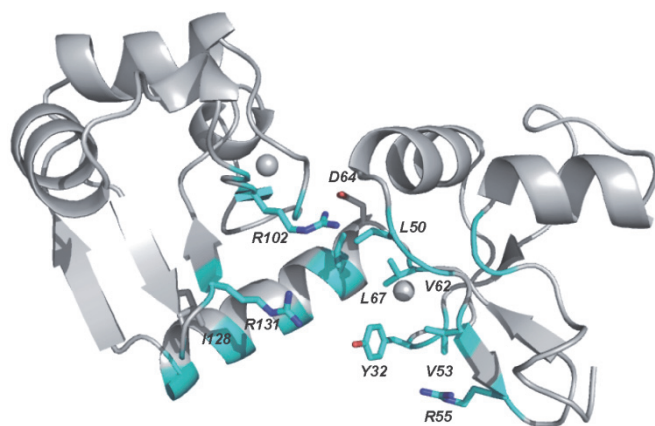

Supplement: Figure S3 — Projection of the mammalian two-hybrid data for the E6APpep interaction on the E6 structure. Cyan: residues identified by x-ray analysis to form the interaction domain for E6APpep [12]. Side chains of residues that are required for E6APpep binding, as revealed by mutational analyses in mammalian two-hybrid assays, are indicated. (PDF) [file pone.0112514.s003.pdf]
