# Supplementary material for: The E6AP Binding Pocket of the HPV16 E6 Oncoprotein Provides a Docking Site for a Small Inhibitory Peptide Unrelated to E6AP, Indicating Druggability of E6
Source: PLoS One. 2014 Nov 10;9(11):e112514. doi: 10.1371/journal.pone.0112514 (PMC4226571; doi:10.1371/journal.pone.0112514)
Supplement: Table S1 — Mutations reducing intracellular binding of HPV16 E6 to E6APpep or pep11**. The location of HPV16 E6 amino acid residues in the HPV16 E6 structure and their involvement in E6APpep binding according to x-ray data [12] are listed. For mammalian two-hybrid analyses, luciferase values for the interaction of wildtype HPV16 E6 with E6APpep or pep11**, respectively, were set at 100%. Shown are relative luciferase activities (RLA) in percent upon mutation of the indicated amino acid residues. Mutations resulting in an over 1.5-fold reduction are highlighted in bold. Standard deviations are indicated. * : within the E6N self-association interface [19]. (DOCX) [file pone.0112514.s004.docx]

**Table S1.** Mutations reducing intracellular binding of HPV16 E6 to E6APpep or pep11**.

| **res.** | **location on E6**  **(x-ray)** | **x-ray** | **mut.** | **intracellular binding analyses (mammalian two-hybrid)** | |
| --- | --- | --- | --- | --- | --- |
|  |  | **involved in E6APpep binding** |  | **E6APpep binding**  **(RLA in %)** | **pep11** binding**  **(RLA in %)** |
| **I23** | **surface** | **no** | **I23Q** | **67,1 ± 7,6** | **71,0 ± 4,7** |
| **H24** | **surface** | **no** | **H24Q** | **88,1 ± 5,7** | **75,0 ± 2,5** |
|  |  |  | **H24R** | **80,1 ±12,2** | **56,7 ± 5,1** |
| **Y32** | **within E6AP b.p.** | **yes** | **Y32A** | **53,5 ± 4,5** | **7,6 ± 0,4** |
| **K34** | **surface** | **no** | **K34E** | **75,3 ± 4,8** | **117,4 ± 7,2** |
| **Q35** | **surface** | **no** | **Q35A** | **114,8 ±11,4** | **103,0 ±12,3** |
|  |  |  | **Q35R** | **116,3 ± 5,7** | **103,2 ± 8,7** |
| **L38** | **surface** | **no** | **L38E** | **172,9 ±23,0** | **140,9 ± 8,0** |
| **R39** | **surface*** | **no** | **R39A** | **79,1 ± 6,8** | **89,7 ± 2,9** |
| **R40** | **surface*** | **no** | **R40A** | **114,5 ± 8,2** | **117,8 ± 6,8** |
| **D44** | **surface*** | **no** | **D44R** | **67,7 ± 3,9** | **44,6 ± 2,2** |
| **R48** | **close to E6AP b.p.** | **no** | **R48A** | **98,6 ±16,8** | **114,2 ± 5,5** |
| **L50** | **within E6AP b.p.** | **yes** | **L50E** | **41,6 ± 2,1** | **5,4 ± 0,3** |
| **C51** | **within E6AP b.p.** | **yes** | **C51F** | **115,2 ±16,5** | **113,3 ±12,5** |
| **V53** | **within E6AP b.p.** | **yes** | **V53E** | **45,2 ± 4,1** | **7,3 ± 0,9** |
| **R55** | **within E6AP b.p.** | **yes** | **R55A** | **64,2 ± 9,6** | **130,0 ±13,3** |
| **V62** | **within E6AP b.p.** | **yes** | **V62A** | **62,7 ±10,3** | **60,4 ± 3,1** |
| **D64** | **close to E6AP b.p.** | **no** | **D64A** | **65,3 ± 6,6** | **58,4 ± 8,6** |
|  |  |  | **D64H** | **63,1 ± 5,8** | **57,3 ±10,4** |
| **L67** | **within E6AP b.p.** | **yes** | **L67A** | **48,0 ± 2,7** | **46,1 ± 4,7** |
| **Y70** | **within E6AP b.p.** | **yes** | **Y70A** | **99,6 ±10,9** | **57,5 ± 7,8** |
| **S74** | **within E6AP b.p.** | **yes** | **S74R** | **71,5 ±10,6** | **62,7 ± 5,3** |
| **L100** | **within E6AP b.p.** | **yes** | **L100A** | **131,7 ±28,7** | **127,3 ±15,3** |
| **R102** | **within E6AP b.p.** | **yes** | **R102E** | **42,3 ± 4,6** | **10,3 ± 0,3** |
| **N105** | **close to E6AP b.p.** | **no** | **N105A** | **81,3 ±13,4** | **106,9 ±19,6** |
| **Q107** | **within E6AP b.p.** | **yes** | **Q107A** | **79,7 ± 4,9** | **101,1 ±19,7** |
| **K108** | **close to E6AP b.p.** | **no** | **K108A** | **93,7 ±10,8** | **138,1 ±22,9** |
| **L119** | **surface** | **no** | **L119E** | **89,0 ± 9,0** | **100,8 ±15,6** |
| **D120** | **surface** | **no** | **D120A** | **117,2 ±20,2** | **104,7 ±16,1** |
|  |  |  | **D120K** | **104,9 ± 3,8** | **90,8 ± 7,5** |
| **K121** | **surface** | **no** | **K121A** | **124,3 ± 4,0** | **168,3 ±25,5** |
| **K122** | **surface** | **no** | **K122A** | **111,8 ± 3,9** | **167,5 ±17,6** |
| **H126** | **surface** | **no** | **H126A** | **80,9 ±16,9** | **109,7 ±14,5** |
|  |  |  | **H126E** | **63,0 ± 4,0** | **98,7 ±11,8** |
| **I128** | **within E6AP b.p.** | **no** | **I128A** | **52,0 ± 3,4** | **18,0 ± 0,9** |
|  |  |  | **I128E** | **47,8 ± 2,1** | **10,8 ± 0,3** |
| **R129** | **within E6AP b.p.** | **yes** | **R129A** | **291,3 ±38,0** | **190,2 ±18,4** |
|  |  |  | **R129E** | **111,0 ±17,3** | **119,9 ±12,1** |
| **R131** | **within E6AP b.p.** | **yes** | **R131E** | **40,9 ± 7,2** | **48,9 ± 1,8** |
| **T133** | **close to E6AP b.p.** | **no** | **T133A** | **151,1 ±21,0** | **135,2 ±26,2** |
| **C140** | **surface** | **no** | **C140E** | **142,5 ±21,2** | **145,9 ± 1,5** |
